# Demenz mit Lewy-Körperchen

**DOI:** 10.1055/a-2791-7810

**Published:** 2026-06-24

**Authors:** Lena Marth, Adèlina Lund, Günter Höglinger, Johannes Levin

**Affiliations:** 1 Neurologische Klinik, LMU Klinikum, Ludwigs-Maximilians-Universität München (LMU), München, Germany; 2 Deutsches Zentrum für Neurodegenerative Erkrankungen (DZNE), München, Germany; 3 Munich Cluster for Systems Neurology (SyNergy), München, Germany

**Keywords:** DLB, Demenz, alpha-Synuklein, atypische Parkinson-Syndrome, Demenz mit Lewy-Körperchen, Lewy-Körperchen, DLB, dementia, Lewy bodies, alpha-synuclein, atypical parkinsonism, Dementia with Lewy bodies

## Abstract

Die Demenz mit Lewy-Körperchen (DLB) ist nach der Alzheimer-Demenz die zweithäufigste neurodegenerative Demenzform und zählt zu den atypischen Parkinson-Syndromen. Sie ist charakterisiert durch kognitive Fluktuationen, visuelle Halluzinationen, eine REM-Schlaf-Verhaltensstörung und Parkinson-Symptome. Typischerweise treten die kognitiven Einbußen vor oder bis maximal ein Jahr nach Beginn der motorischen Symptomatik auf („Einjahreskriterium“). Neuropathologisch liegt eine α-Synukleinopathie mit Lewy-Körperchen in Neuronen vor, häufig begleitet von Kopathologien. Die Diagnosestellung erfolgt klinisch nach den McKeith-Kriterien unter Einbeziehung z.B. bildgebender Biomarker. Therapeutisch stehen symptomorientierte Ansätze im Vordergrund. Krankheitsmodifizierende Therapien stehen bislang nicht zur Verfügung, werden jedoch in Therapiestudien erprobt. Die Entwicklung biologisch definierter Diagnosekriterien und molekularer Biomarker ist zentral für zukünftige Therapiestudien.

## Abstract (en)

Dementia with Lewy bodies (DLB) is the second most common neurodegenerative type of dementia after Alzheimer’s disease and belongs to the atypical parkinsonian syndromes. It is characterized by cognitive fluctuations, visual hallucinations, REM sleep behavior disorder, and parkinsonism. Typically, cognitive impairment appears before or within one year after the onset of motor symptoms (“one-year rule”). Neuropathologically, DLB is an α-synucleinopathy with neuronal Lewy bodies, often accompanied by copathologies. Diagnosis is based on clinical McKeith criteria and often supported by imaging biomarkers. Treatment is mainly symptomatic. Disease-modifying therapies are not yet available but are being investigated in clinical trials. The development of biologically defined diagnostic criteria and molecular biomarkers is crucial for future therapeutic studies.

## Klinische Krankheitsdefinition

Die Demenz mit Lewy-Körperchen (DLB, Abk. für Engl.: Dementia with Lewy bodies) ist eine neurodegenerative Erkrankung, die zu den atypischen Parkinson-Syndromen gezählt wird. Sie ist charakterisiert durch eine früh im Krankheitsverlauf auftretende Demenz mit meist fluktuierenden kognitiven Leistungen, einem dysexekutiven neuropsychologischen Profil mit visuokonstruktiven Defiziten und häufig ausgeschmückten visuellen Halluzinationen. Darüber hinaus entwickeln viele Betroffene (>85%) eine Parkinson-Symptomatik und zeigen bereits Jahre vor Einsetzen der demenziellen Entwicklung eine REM-Schlaf-Verhaltensstörung (RBD, Abk. für Engl.: REM sleep behavior disorder). Nicht selten sind auch begleitende vegetative Störungen 1 . Typischerweise treten die kognitiven Einbußen bei der DLB bereits vor oder bis maximal ein Jahr nach Beginn der motorischen Symptomatik auf, was sie klinisch von der Parkinson-Demenz (PDD, Abk. für Engl.: Parkinson‘s disease dementia) abgrenzt. Man nennt diese Abgrenzung auch das „Einjahreskriterium“. Der Verlauf der DLB ist mit einer durchschnittlichen Überlebenszeit von lediglich 6–8 Jahren ab Symptombeginn ungünstig. Wie die Parkinson-Krankheit (PD, Abk. für Engl.: Parkinson’s disease) und die PDD gehört die DLB zu den α-Synukleinopathien vom Lewy-Typ, welche durch eine charakteristische intrazelluläre Aggregation des Proteins α-Synuklein (α-Syn) in Form von Lewy-Körperchen (LB, Abk. für Engl.: Lewy bodies) in Neuronen gekennzeichnet sind. Eine schematische Übersicht über die pathophysiologischen und klinischen Kernaspekte der DLB ist im graphischen Steckbrief zusammengefasst ( Abb. 1 ).

**Abb. 1:**
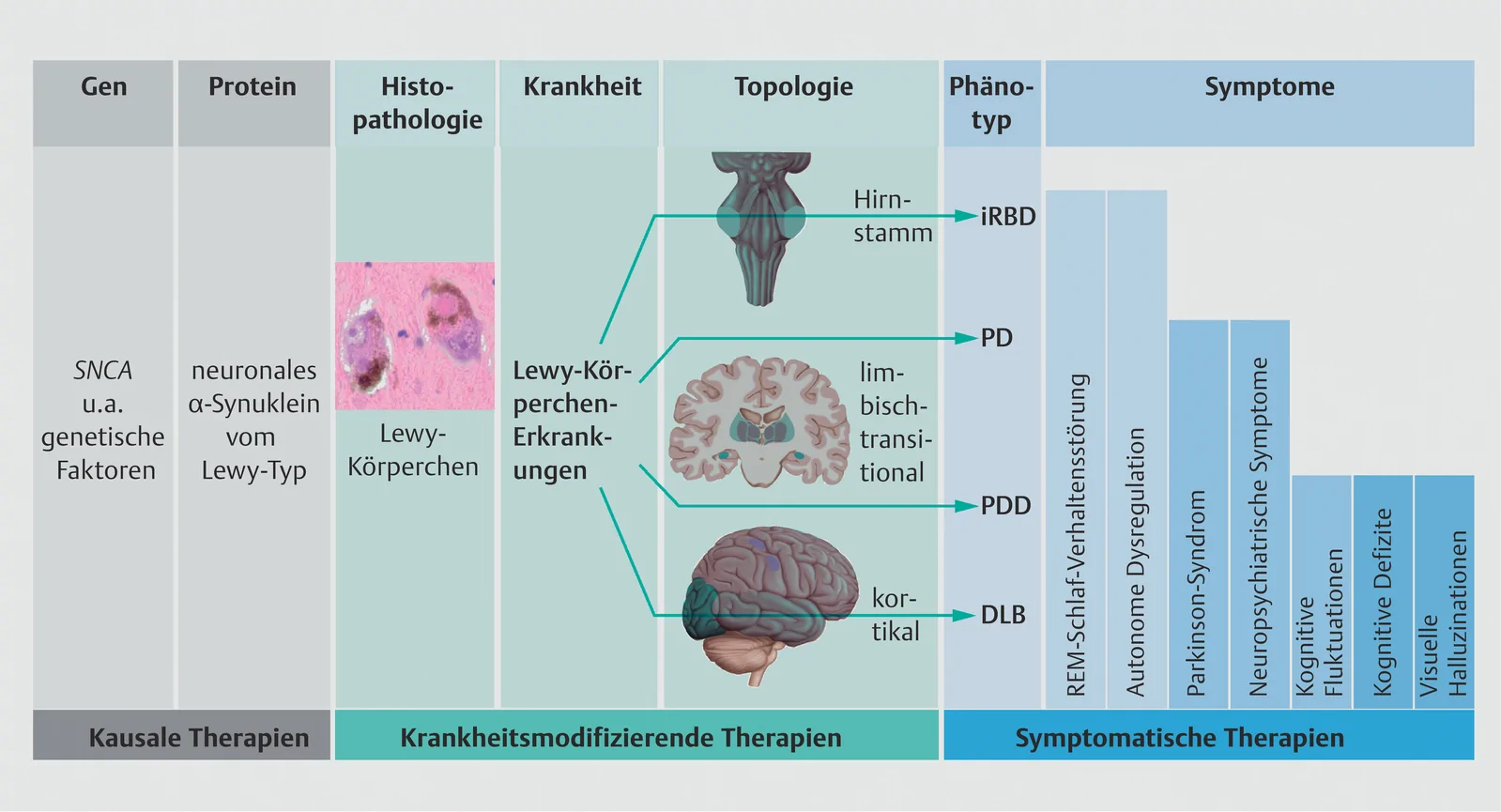
Graphischer Steckbrief Lewy-Körperchen-Erkrankungen. Die schematische Darstellung illustriert die verschiedenen Krankheitsebenen der Lewy-Körperchen-Erkrankungen beginnend bei den biologischen Ursachen, über die Neuropathologie und Topologie bis hin zur klinischen Symptomatik. Kausale und krankheitsmodifizierende Ansätze setzen an der molekularen Pathologie an, symptomatische Therapien können Symptome lindern, haben jedoch keinen Einfluss auf die Krankheitsprogression (rechte Spalte). Andere genetische Faktoren der PD und PDD: PRKN, PINK1, PARK7, LRRK2, VPS35, CHCHD2, GBA1 und GCH1 Gene, 22q11.2 Deletionssyndrom 33 . Andere genetische Faktoren der DLB: GBA1, APOE ε4 Allel. iRBD: isolierte REM-Schlaf-Verhaltensstörung; PD: Parkinson-Krankheit; PDD: Parkinson-Demenz; DLB: Demenz mit Lewy-Körperchen.

## Neuropathologische und biologische Krankheitsdefinition

Makroskopisch ist in Gehirnen von Menschen mit DLB analog zur PD typischerweise eine Depigmentierung der Pars compacta der Substantia nigra (SN) im Mesenzephalon zu finden. Dies ist Ausdruck der Degeneration der dopaminergen Neurone, welche durch ihren hohen Gehalt an Neuromelanin stark pigmentiert sind. Daneben zeigt sich meist eine leichte bis mäßige kortikale Atrophie, welche oftmals geringer ausgeprägt als bei der Alzheimer-Demenz (AD) ist.

Als diagnostischer Goldstandard der DLB gilt weiterhin der histopathologische Nachweis von LB in Somata und Lewy-Neuriten in Fortsätzen von Nervenzellen *post mortem* . Bei den LB handelt es sich überwiegend um Ansammlungen von, zu unverzweigten Filamenten angeordneten, posttranslational modifizierten, α-Syn Aggregaten. Hirnstamm-LBs befinden sich meist in den pigmentierten dopaminergen Neuronen der SN und bestehen aus einem runden eosinophilen Kern umgeben von einem blassen Halo. Neokortikale LBs stellen sich hingegen morphologisch heterogener und oft ohne Halo dar 2 .

Das neuropathologische Staging der LB-Pathologie erfolgt meist anhand der Newcastle-McKeith Kriterien des DLB-Konsortiums 1 . Diese klassifizieren die α-Syn-Pathologie nach ihrem Verteilungsmuster in hirnstammbetont, limbisch-transitional oder diffus neokortikal und beziehen eine begleitende AD-Kopathologie in eine wahrscheinlichkeitsskalierte Diagnose ein. Neuropathologisch wird eine LB-Erkrankung diagnostiziert, ohne die klinischen Entitäten PD und DLB voneinander abzugrenzen, sodass es sich neuropathologisch um eine Entität handelt. Die ebenfalls für die DLB gebräuchlichen Braak-Stadien hingegen beschreiben ein hypothetisches Progressionsmodell, bei dem sich die LB-Pathologie von den dorsalen Vaguskernen und dem Bulbus olfactorius über Mittelhirn und limbische Strukturen bis in den Neokortex ausbreitet 3 .

Bei neurodegenerativen Erkrankungen wird die klinische Präsentation maßgeblich durch die Lokalisation der molekularen Kernpathologie bestimmt. Es ist daher essenziell, zwischen dem klinischen Syndrom und der zugrundeliegenden Pathologie zu unterscheiden. Beispielsweise führen im Fall der AD die Tau-Ablagerungen und die damit verbundene Neurodegeneration im mesialen Temporallappen (mTL) typischerweise zu einem amnestischen Syndrom. Liegt die Tau-Pathologie jedoch vorwiegend in anderen Hirnarealen vor, kann es zu atypischen Präsentationen, wie z. B. einem kortikobasalen Syndrom, trotz ursächlicher AD-typischer Tau-Pathologie kommen. Im Fall der DLB bedingt die okzipital betonte Verteilung der LB-Pathologie wesentlich die charakteristischen visuokonstruktiven Defizite. Gleichwohl erklärt diese topographische Verteilung nur einen Teil des komplexen klinischen Bildes, was maßgeblich auf die häufig vorliegenden Kopathologien zurückzuführen ist. Autopsiebefunde mit reiner LB-Pathologie sind insgesamt in der Minderheit. 51–73% der DLB-Fälle weisen bei Autopsie ein deutliches Ausmaß an beta-Amyloid (Aβ)-Plaques und Tau-Tangles auf, sodass neben der DLB auch die Kriterien einer AD-Pathologie erfüllt werden. Darüber hinaus ergab eine Meta-Analyse, dass etwa 68% der DLB-Patienten im Amyloid-PET positiv sind 4 . Patienten mit DLB und AD-Kopathologie neigen zu einem schnelleren kognitiven und funktionellen Abbau und einer höheren Mortalität, als solche mit einer reinen LB-Pathologie 5 . Darüber hinaus zeigen DLB-Patienten mit AD-Kopathologie eine deutlichere Atrophie des mTL und entwickeln seltener visuelle Halluzinationen oder Parkinson-Symptome. Zudem wirft dies die auf histopathologischer Ebene ungelöste Frage auf, wo die Grenzen zwischen DLB sensu strictu, DLB mit AD-Kopathologie und AD mit LB-Kopathologie verläuft, bzw. ob es überhaupt eine klar definierte Grenze gibt oder es sich vielmehr um ein kontinuierliches Krankheitsspektrum handelt ( Abb. 2 ). Die Prävalenz einer zusätzlichen *Transactive response DNA-binding protein 43* (TDP-43)-Pathologie, wie sie bei der frontotemporalen Lobärdegeneration (FTLD) vorkommen kann, liegt bei bis zu 56% 2 6 . Hierbei sind vor allem limbische Strukturen früh betroffen und eine TDP-43-Kopathologie ist mit einer stärkeren kognitiven Beeinträchtigung sowie einer ausgeprägteren Atrophie des mTL assoziiert 2 . In einer klinisch-neuropathologischen Studie zeigte sich außerdem in 56,4% der DLB-Fälle eine zusätzliche *aging-related tau astrogliopathy* (ARTAG)-Pathologie mit Schwerpunkt im mTL 7 . Die klinische Bedeutung einer gleichzeitig vorliegenden LB- und ARTAG-Pathologie ist bislang unzureichend erforscht, jedoch gibt es Hinweise für ein Zusammenwirken beider Pathologien zu einem synergistischen Krankheitseffekt mit stärkeren kognitiven Einbußen und ausgeprägterer Hirnatrophie 8 . Darüber hinaus liegt in etwa 50% der durch Autopsie bestätigten DLB-Fälle zusätzlich eine relevante vaskuläre Beteiligung vor 9 .

**Abb. 2:**
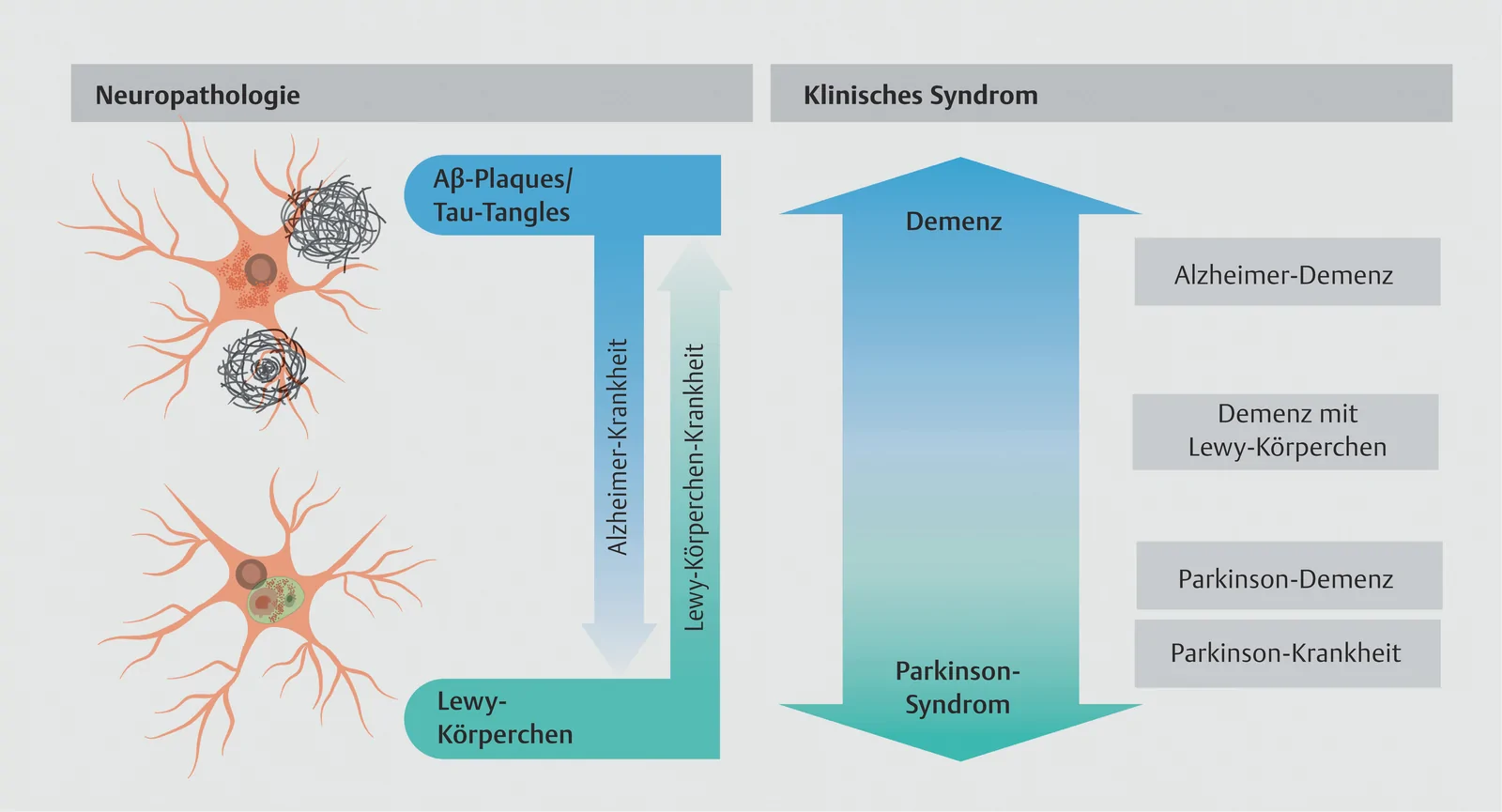
Schematische Darstellung des neuropathologischen Kontinuums zwischen Alzheimer- und Lewy-Körperchen-Erkrankungen. Misch- und Kopathologien aus einer AD- (rot: β-Amyloid-Plaques, Tau-Tangles) und einer LB-Pathologie (blau: Lewy-Körperchen) sind sehr häufig. Eine klare neuropathologische Trennung ist nicht immer möglich, sodass es sich um ein neuropathologisches Kontinuum handelt. Die Häufigkeit von Kopathologien variiert je nach klinischer Diagnose. Am häufigsten ist eine AD-Kopathologie bei der DLB, gefolgt von der PDD und der PD. Auch bei der AD kann eine LB-Kopathologie vorliegen 41 .

## Epidemiologie

Die Epidemiologie der DLB ist kompliziert. Kernursachen sind wie oben beschrieben, dass die LB-Kopathologie der AD häufig ist, und die DLB mit AD-Kopathologie zum Teil schwierig zu diagnostizieren ist. Die klinische Erfahrung zeigt, dass das klassische klinische Bild mit Demenz, Fluktuationen der Kognition und Wachheit, visuellen Halluzinationen und Parkinson-Symptomen vergleichsweise selten ist. Dem gegenüber ist eine LB-(Ko-) Pathologie bei Demenzen vergleichsweise häufig. Der aktuellen Literatur zufolge ist die DLB nach der AD die zweithäufigste neurodegenerative Demenzform. Die Inzidenz liegt bei etwa 0,5–1,6 Fälle pro 1000 Personenjahre. Typischerweise manifestiert sich die Erkrankung im Mittel mit 70–75 Jahren, Fälle mit einem Krankheitsbeginn<65 Jahre sind nur selten beschrieben 2 . In bevölkerungsbasierten Studien wird die Prävalenz der DLB in älteren Bevölkerungsgruppen auf 4–7% geschätzt 10 . In einer neuropathologischen Studie konnte bei 5% der älteren Personen eine reine DLB-Pathologie und in 10% eine DLB-Pathologie mit AD-Kopathologie identifiziert werden 11 . Je nach Studie liegt Demenzen in bis zu 15% der Fälle eine DLB zugrunde. In neuropathologischen Studien kann sogar in bis zu 20% der Demenz-Fälle eine ursächliche DLB identifiziert werden 10 , was die Komplexität der Datenlage verdeutlicht und auch, dass die DLB mangels geeigneter Biomarker sowie der häufig vorliegenden AD-Kopathologie vermutlich deutlich unterdiagnostiziert wird. Männer scheinen etwas häufiger betroffen zu sein als Frauen.

## Ätiopathogenese

Die Ätiologie der DLB gilt als multifaktoriell und ist noch unzureichend geklärt. Angenommen wird ein Zusammenspiel von genetischen, molekularen und umweltbedingten Faktoren, die zur pathologischen Aggregation von α-Syn und zur Neurodegeneration führen. Der genaue Auslöser für die Fehlfaltung von α-Syn bei sporadischen Erkrankungen bleibt bislang unklar, jedoch erscheinen verschiedene Mechanismen beteiligt zu sein, darunter oxidativer Stress, gestörter Proteasomen- und Autophagieabbau, mitochondriale Dysfunktion und Inflammation. Von genetisch bedingten Synukleinopathien ist bekannt, dass die α-Syn-Gendosis und pathogene Varianten im α-Syn-Gen zu aggregationsfördernden Veränderungen der Aminosäuresequenz führen. Obwohl die Aggregation von α-Syn eine zentrale Rolle in der Pathogenese der DLB zu spielen scheint, ist die Relevanz der ausgereiften LBs für die Ausbildung klinischer Symptome unklar. Wahrscheinlicher ist, dass oligomere Aggregate die wesentliche toxische Spezies sind 2 . Die sogenannte „Aggresom-Hypothese“ postuliert, dass LBs gegebenenfalls im Kontext der schädlichen Aggregationsprozesse neben ihrer Bedeutung in der Pathologie auch protektiv sein könnten, indem sie toxische Oligomere binden. Ein Argument für diese Hypothese ist, dass histopathologisch beobachtet wurde, dass Nervenzellen mit vollständig ausgereiften LBs weniger zu Apoptose neigten als solche mit kleineren Aggregatspezies 2 .

Als etablierter Risikofaktor für eine DLB gilt eine auffällige Familienanamnese. Geschwister von Betroffenen haben ein 2,3-fach erhöhtes Risiko ebenfalls zu erkranken. Die große Mehrzahl der DLB-Fälle ist jedoch sporadisch, tritt also nicht familiär gehäuft auf. Bei den familiären Fällen gelten Varianten mit sehr hoher Penetranz als äußerst selten. Es bestehen einige Fallserien über Familien mit seltenen Punktmutationen im *SNCA* -Gen, wobei häufig ein prädominanter PD-Phänotyp vorliegt, sodass die Konsensus-Kriterien der DLB nicht erfüllt werden. Auch seltene homozygote oder compound-heterozygote Varianten im *Vacuolar Protein Sorting 13 homolog C* ( *VPS13C* ) Gen konnten in neuropathologisch bestätigten DLB-Fällen nachgewiesen werden und scheinen mit dem Auftreten einer DLB assoziiert zu sein 12 . Bis heute wurden jedoch keine hoch penetranten Varianten identifiziert, die eine DLB bedingen. Vielmehr ist von unterschiedlich starken genetischen Risikofaktoren auszugehen. Mittels genomweiter Assoziations-Studien konnten die Apolipoprotein E ε4 (APOE ε4), *SNCA* , und Glukozerebrosidase ( *GBA* ) Gene wiederholt als Risikoloci bestätigt werden 13 . Homo- und auch in geringerem Ausmaß heterozygote Träger des APOE ε4 Allels entwickeln häufig eine gemischte DLB-AD-Pathologie, das APOE ε4 Allel ist jedoch auch bei der reinen DLB überrepräsentiert. Interessanterweise konnte keine Assoziation des APOE ε4 Allels mit der PD identifiziert werden. Auch die Assoziation mit dem *GBA* -Gen ist gut etabliert, Menschen mit DLB tragen mit 8-fach erhöhter Wahrscheinlichkeit im Vergleich zu gesunden Kontrollen eine prädisponierende *GBA* -Variante 13 .

## Aktuelle Diagnosekriterien

Klinisch wird die Diagnose einer DLB nach den Konsensus-Kriterien des DLB-Konsortiums nach McKeith gestellt ( Abb. 3 ) 1 . Eingangskriterium ist eine erworbene und alltagsrelevante kognitive Störung. Diese sollte bereits früh im Krankheitsverlauf, vor oder bis maximal ein Jahr nach Auftreten motorischer Symptome, bestehen 1 . Die Diagnose einer wahrscheinlichen DLB kann gestellt werden, wenn zusätzlich entweder 2 klinische Haupt-Symptome ( *core-symptoms* ) oder ein klinisches Haupt-Symptom und ein indikativer Biomarker vorliegen. Eine mögliche DLB kann diagnostiziert werden, wenn entweder ein klinisches Haupt-Symptom oder ein indikativer Biomarker vorliegen. Eine umfassende neuropathologische Validierung der McKeith-Kriterien ist ausstehend.

**Abb. 3:**
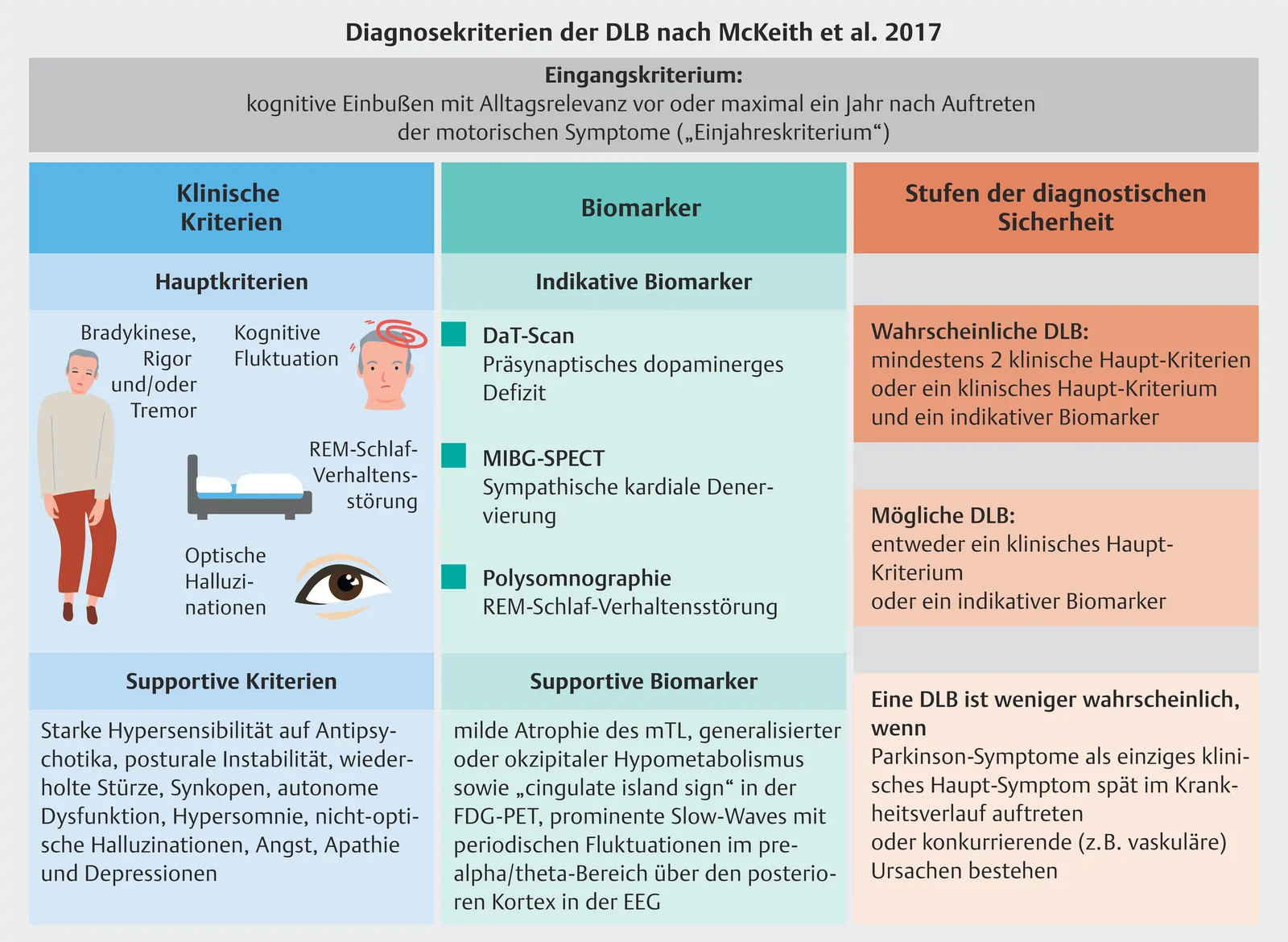
Diagnosekriterien der DLB nach den Kriterien des DLB-Konsortiums nach McKeith et al. 2017 1 .

### Klinische Symptomatik

#### Klinische Haupt-Symptome

- Fluktuationen der Kognition und Wachheit treten typischerweise früh im Krankheitsverlauf auf, sind jedoch häufig nicht trivial zu erfragen. Am verlässlichsten scheint es zu sein nach spontanen Veränderungen der Kognition, Tagesschläfrigkeit, „in die Umgebung Starren“ oder Episoden inkohärenter Sprache zu fragen 1 .
- Eine RBD tritt bei bis zu 76% der DLB-Patienten auf. Hierbei handelt es sich um eine Parasomnie, bei der Trauminhalte aufgrund einer ausbleibenden Atonie in der REM-Schlaf-Phase motorisch ausgelebt werden. Besonders wahrscheinlich ist eine RBD, wenn es sich bei den Trauminhalten um lebhafte Verfolgungs- oder Angriffssituationen handelt oder der Bettpartner bereits Verletzungen davongetragen hat 1 .
- Visuelle Halluzinationen sind typischerweise sehr ausgeschmückt und umfassen Menschen, Tiere und Gegenstände. In bis zu 80% der DLB-Fälle treten visuelle Halluzinationen im Krankheitsverlauf auf 1 . Diese können entweder im Zusammenhang mit der dopaminergen Medikation, aber auch unabhängig davon auftreten.
- Parkinson-Symptome treten bei etwa 85% der Patienten mit DLB im Krankheitsverlauf auf, sind jedoch meist subtiler ausgeprägt als bei der PD und werden deshalb leichter verkannt 1 . Im Gegensatz zur PD genügt bei der DLB bereits ein klinisches Parkinson-Symptom, also Rigor, Tremor oder Bradykinese, um das Kriterium einer motorischen Symptomatik zu erfüllen.

#### Unterstützende klinische Symptomatik

Neben den klinischen Haupt-Symptomen gibt es weitere Symptome, die die Diagnose einer DLB unterstützen können, jedoch nicht ausreichend spezifisch sind, um als klinisches Haupt-Kriterium gewertet zu werden. Hierzu zählt eine starke Hypersensibilität auf „klassische“ Antipsychotika, welche bei Patienten mit DLB meist deutlich ausgeprägter als bei PD ist und zu irreversiblen Schäden (u. a. akinetische Krise, Vigilanzminderungen bis hin zum Tod) führen kann. Ebenfalls zu den unterstützenden klinischen Merkmalen zählen eine posturale Instabilität, wiederholte Stürze, Synkopen, autonome Dysfunktion (orthostatische Dysregulation, Obstipationen, Blasenstörung), Hypersomnie, nicht-optische Halluzinationen und neuropsychiatrische Symptome wie Angst, Apathie und Depressionen 1 .

### Biomarker

#### Indikative Biomarker

- Dopamintransporter-Szintigraphie mit^123^Iod FP-CIT SPECT (DaT-Scan): Der Nachweis einer reduzierten Dichte des präsynaptischen Dopamintransporters mittels DaT-Scan ist gut etabliert und erreicht eine Sensitivität von 78% und Spezifität von bis zu 90% in der Unterscheidung von AD und DLB 1 . Zur Differenzialdiagnose der DLB zur PD, FTLD oder anderen atypischen Parkinson-Syndromen ist das DaT-Scan nicht geeignet, da diese ebenfalls mit einer Degeneration der dopaminergen Neurone einhergehen können.
- ^123^Iod-MIBG Myokard-Szintigraphie (MIBG-SPECT) : Mittels MIBG-SPECT kann die postganglionäre sympathische kardiale Innervation quantifiziert werden, welche bei der DLB im Gegensatz zur AD typischerweise reduziert ist. Bei der Unterscheidung beider Erkrankungen erreicht das MIBG-SPECT eine Sensitivität von bis zu 77% und eine Spezifität von bis zu 94% 1 . Komorbiditäten wie eine Polyneuropathie oder ischämische Herzkrankheit können jedoch zu einer Verfälschung der Ergebnisse führen.
- Polysomnographie (PSG): Der Nachweis einer RBD mittels PSG ist ein sehr spezifischer Prädiktor einer α‑Synukleinopathie. Im Falle eines mittels PSG bestätigten REM-Schlafs ohne Atonie bei Personen mit Demenz und anamnestischen Hinweisen auf eine RBD liegt die Wahrscheinlichkeit einer zugrundeliegenden α‑Synukleinopathie bei>90% 1 .

#### Supportive Biomarker

Supportive Biomarker können die Diagnose einer DLB unterstützen, sind jedoch nicht spezifisch genug, um als indikative Biomarker in die Diagnosekriterien einzugehen. Eher für eine DLB und gegen eine AD spricht eine im Vergleich zur AD weniger stark ausgeprägte Atrophie des mTL, insbesondere der Hippocampi. Auch ein generalisierter oder okzipitaler Hypometabolismus sowie die relative Aussparung der posterioren Cingulums („cingulate island sign“) in der FDG-PET können die Diagnose einer DLB unterstützen. Weiterhin typisch für die DLB sind prominente Slow-Waves mit periodischen Fluktuationen im pre-alpha/theta-Bereich über dem posterioren Kortex in der EEG 1 .

## Molekulare Biomarker

Aufgrund der heterogenen klinischen Verläufe sowie der hohen Rate an zerebralen Kopathologien stellt die Diagnosestellung der DLB weiterhin eine Herausforderung dar. Aus neuropathologischen Studien ist bekannt, dass in vielen Demenz-Fällen zeitgleich eine LB- sowie eine AD-Pathologie vorliegt und es teils auch mittels neuropathologischer Aufarbeitung nicht sicher möglich ist, die symptombestimmende Kernpathologie sicher zu benennen. *In vivo* erhalten viele dieser Patienten die Diagnose einer reinen AD, da für die Aβ-Pathologie breit verfügbare Biomarker vorliegen. Dies unterstreicht den Bedarf an molekularen Biomarkern, welche die LB-Pathologie zuverlässig abbilden können, und die Notwendigkeit biologisch-basierter Diagnosekriterien analog zu den A/T/N-Kriterien (Amyloid/Tau/Neurodegeneration) des *National Institute on Aging and Alzheimer’s Association* (NIA-AA) für die AD 14 .

Molekulare Biomarker für eine α‑Synukleinopathie vom Lewy-Typ befinden sich zwar in Entwicklung, sind jedoch für die Verwendung in der klinischen Routine noch nicht zugelassen. Zu nennen ist insbesondere der *α-Syn Seed Amplification Assay* (α-Syn-SAA) aus Liquor. Dieser hat bei reiner DLB-Pathologie das Potenzial die Diagnose zukünftig molekular zu sichern. Aufgrund der häufigen Mischpathologien wird jedoch eine Kombination mehrerer molekularer Biomarker nötig sein, um die Erkrankung auf ätiologischer Ebene präzise zu charakterisieren. Molekulare Biomarker für eine Aβ-Pathologie sind breit verfügbar, gut validiert und erreichen hohe diagnostische Genauigkeit. Aufgrund der hohen Prävalenz einer Aβ- oder AD-Kopathologie von etwa 50–70% bei der DLB ( Abb. 2 ) haben diese Marker jedoch nur ein geringes Potenzial eine DLB von einer AD zu unterscheiden. Auch der α-Syn-SAA kommt in diesen Fällen an seine diagnostischen Grenzen, da eine Vielzahl der Patienten mit sporadischer AD ebenfalls eine α-Syn-Kopathologie aufweist 14 . Bei gleichzeitigem Vorliegen einer Aβ- und einer α‑Syn-Pathologie stellt sich die Frage, welche der beiden Pathologien führend für die klinische Symptomatik ist. In solchen Fällen kann die Bildgebung mittels Tau-PET hilfreich sein, welche zuverlässig die fibrilläre Tau-Pathologie im Rahmen einer AD in Neuronen widerspiegelt. Hierfür weitgehend ungeeignet sind die aktuell verfügbaren, fluiden Tau-Biomarker, inklusive p-Tau181, da diese bereits bei einer Aβ-Pathologie ansprechen und keinen sicheren Nachweis einer fibrillären Tau-Pathologie darstellen 15 . Ein Patient, welcher im Liquor sowohl eine Aβ- als auch α‑Syn-Pathologie hat, im Tau-PET jedoch keine gesteigerte Tracer-Aufnahme aufweist, leidet sehr wahrscheinlich an einer DLB mit Aβ-Kopathologie.

Eine genetische Testung wird derzeit aufgrund des geringen diagnostischen Mehrwerts bei der DLB nicht routinemäßig empfohlen. Genetische Risikofaktoren der DLB überlappen signifikant sowohl mit denen der AD als auch der PD. Monogenetische Ursachen der DLB sind äußerst selten.

## Therapie

Die Therapie der DLB gestaltet sich aufgrund der vielseitigen klinischen Manifestationen im Spektrum zwischen Demenz und Bewegungsstörung oft komplex und beschränkt sich auf symptomatische Behandlungsansätze. Krankheitsmodifizierende Therapien stehen derzeit nicht zur Verfügung. Auch für die Wirksamkeit symptomatischer Therapieansätze besteht eine begrenzte Evidenz. Die Indikationsstellung sollte symptomorientiert und Patienten-zentriert erfolgen. Optimal erscheint eine Kombination aus pharmakologischen und supportiven Maßnahmen inklusive Physio- und Ergotherapie sowie Logopädie ( Tab. 1 ).

**Table:** **Tab. 1** Symptomatische Therapien der DLB.

Symptomkomplex	Supportive Maßnahmen	Medikamentöse Maßnahmen	Hinweise
**Motorische Symptome**	Physiotherapie	Levodopa (z. B. 50 mg 3×täglich, bei guter Verträglichkeit Steigerung bis zur adäquaten Symptomkontrolle möglich)	Geringeres Ansprechen als bei PD; „ *start low, go slow* “; Dopaminagonisten wegen erhöhtem Risiko für Halluzinationen und Tagesmüdigkeit vermeiden
**Kognitive Symptome**	Ergotherapie	AChE-I (Donepezil 10 mg 1×täglich, Rivastigmin bis zu 6 mg 2×täglich oder transdermal 13,3 mg/24 h)	Off-label, leitliniengerecht
**Psychotische Symptome/Halluzinationen**	Überprüfung der Medikation, ggf. absetzen oder Reduktion möglicher auslösender Präparate	Clozapin (z. B. 12,5–50 mg tägl.), Quetiapin (z. B. 25–100mg tägl.)	„Klassische“ Antipsychotika wie Haloperidol oder Risperidon sind kontraindiziert aufgrund des Risikos einer akinetischen Krise
**Angst/Depression**	Psychotherapie	SSRIs/SNRIs, z. B. Sertralin (50–100 mg tägl.) oder Venlafaxin (37,5–75 mg tägl.)	Präparate mit anticholinerger Wirkung (z. B. Amitriptylin) aufgrund potenzieller kognitiver Nebenwirkungen vermeiden
**REM-Schlaf-Verhaltensstörung**		Clonazepam (0,5–2 mg); Melatonin (4 mg retard)	Therapie nur bei erhöhter Verletzungsgefahr oder deutlichem Leidensdruck, z. B. durch gestörten Schlaf, nötig
**Orthostatische Hypotension**	Kompressionsstrümpfe, elastische Bauchbinde, erhöhte Trinkmenge, gesteigerte Kochsalzzufuhr, Gegendruckmanöver	Midodrin (z. B. 2,5 mg 2–3×täglich), Fludrocortison (0,1–0,2 mg täglich)	
**Blasenstörung/imperativer Harndrang**		β3-Agonisten wie z. B. Vibegron (75 mg 1×tägl.) oder Mirabegron (50 mg 1×tägl.)	Urologische Mitbeurteilung, bei Therapieversagen und hohem Leidensdruck intensivierte Therapien wie Botulinumtoxin-Injektionen in den M. Detrusor erwägen
**Obstipationen**	Flüssigkeitszufuhr erhöhen, Mobilisierung, ballaststoffreiche Ernährung, falls vorhanden Anticholinergika und Opiate absetzen	Osmotische Laxantien wie z. B. Macrogol, wenn nicht ausreichend stimulierende Laxantien wie z. B. Bisacodyl	

### Motorische Symptome

können bei klinisch relevanter Ausprägung mittels Levodopa adressiert werden, jedoch ist, wie bei allen atypischen Parkinson-Syndromen, in vielen Fällen ein geringeres Ansprechen als bei der PD zu erwarten. Die Eindosierung sollte vorsichtig nach der Devise „ *start low, go slow* “ erfolgen (z. B. 50mg 3x täglich), da mit einer Häufung psychotischer Symptome gerechnet werden muss. Bei guter Verträglichkeit können auch höhere Tagesdosen angestrebt werden. Von Dopaminagonisten sollte, aufgrund des erhöhten Risikos von Halluzinationen und vermehrter Tagesmüdigkeit, abgesehen werden.

### Kognitive Symptome

können bei leichter bis mittelschwerer DLB Off-label mittels Acetylcholinesterase-Inhibitoren (AChE-I) behandelt werden. Trotz fehlender Zulassung ist die Therapie weit verbreitet und leitliniengerecht. Die höchste Evidenz für eine Wirksamkeit besteht für Donepezil (10 mg 1x täglich) 16 . Auch der Einsatz von Rivastigmin (z. B. 6 mg morgens und abends oder transdermal 13,3 mg/24h) ist in der klinischen Praxis weit verbreitet. Es wird angenommen, dass AChE-I bei der DLB sogar besser wirksam sein könnten, als bei der AD 17 .

### Psychiatrische Symptome

Psychotische Symptome treten bei bis zu 80% der DLB-Patienten auf und sollten im Fall einer pharmakologischen Behandlungsbedürftigkeit laut aktueller Leitlinie zunächst mittels Clozapin therapiert werden 18 . In der klinischen Praxis hingegen wird Quetiapin meist bevorzugt eingesetzt, da es gut verträglich ist. Im Gegensatz zu Clozapin sind bei Quetiapin keine regelmäßigen Blutbildkontrollen zum Ausschluss einer Agranulozytose und kein kardiales Monitoring nötig. Weder für Quetiapin noch für Olanzapin konnte jedoch eine Überlegenheit bei der Behandlung von manifestem Wahn oder Halluzinationen gegenüber Placebo bei DLB-Patienten nachgewiesen werden 18 . Aufgrund der Überempfindlichkeit gegenüber antipsychotischer Medikation ist vom Einsatz älterer „klassischer“ Antipsychotika wie Haloperidol oder Risperidon, unbedingt abzusehen. Diese können zu schweren, teils irreversiblen Schäden, von einer massiven motorischen Verschlechterung (akinetische Krise) über eine starke Sedierung bis hin zum Tod führen. Auch können optische Halluzinationen eine Indikation für den probatorischen Einsatz von AChE-I darstellen. Die Datenlage ist diesbezüglich jedoch uneindeutig 18 . Depressive Verstimmungen sollten zum Erhalt der Lebensqualität bei entsprechender Ausprägung mittels Selektiver Serotonin/Noradrenalin (SNRI)- oder Serotonin-Wiederaufnahmehemmer (SSRI), wie z. B. Venlafaxin oder Sertralin, behandelt werden. Von Präparaten mit anticholinerger Wirkung, wie z. B. Amitriptylin, sollte, aufgrund möglicher kognitiver Nebenwirkungen, abgesehen werden. SSRIs können auch bei Angststörungen eingesetzt werden.

### Weitere Symptome

Eine RBD kann zur Reduktion des Verletzungsrisikos sowie zur Verbesserung der Schlafqualität mittels Clonazepam (0,5–2 mg abends) behandelt werden. Auch kann Melatonin (4 mg ret. zur Nacht) als Zweitlinientherapie verordnet werden. Bei relevanter orthostatischer Dysregulation sollten zunächst konservative Maßnahmen, wie etwa das Tragen von Kompressionsstrümpfen oder besser noch einer elastischen Bauchbinde zum „ *Splanchnic Pooling* “, die Erhöhung der täglichen Trinkmenge und die Steigerung der Kochsalzzufuhr empfohlen werden. Bei ausbleibender Wirksamkeit können Midodrin (z. B. 1%ige Tropfen 7 Tropfen 2–3 mal täglich) oder Fludrocortison (0,1–0,2 mg täglich) eingesetzt werden. Eine neurogene Blasenstörung mit imperativem Harndrang kann mittels β3-Agonisten, wie z. B. Mirabegron (50 mg 1x täglich), adressiert werden. Auch hier sollte auf anticholinerge Substanzen verzichtet werden. Bei Obstipationen können zunächst nicht-medikamentöse Ansätze wie eine gesteigerte Flüssigkeitszufuhr, eine Ballaststoffreiche Ernährung (z. B. Ergänzung um Flohsamenschalen) sowie eine vermehrte Mobilisation angewendet werden. Bei nicht ausreichendem Therapieerfolg können osmotische Laxanzien, wie z. B. Macrogol oder Lactulose, eingesetzt werden. Erst bei Therapieversagen sollen stimulierende Laxantien, wie z. B. Bisacodyl, zum Einsatz kommen.

## Aktuelle Forschungsschwerpunkte

Im Bereich der neurodegenerativen Erkrankungen hat sich in den letzten Jahren ein Paradigmenwechsel weg von rein klinischen und hin zu biologisch definierten *in vivo* Diagnosen vollzogen. Weiter beginnt mit der AD als „Vorreiter-Krankheit“ aktuell das Zeitalter der verlaufsmodifizierenden Therapien für neurodegenerative Erkrankungen. Dies eröffnet neue Forschungsfelder und hat entscheidenden Einfluss auf die Schwerpunkte des wissenschaftlichen Erkenntnisbedarfs. Im Folgenden werden zentrale Forschungsfelder in Bezug auf die DLB beleuchtet.

### Ätiologie

Vor dem Hintergrund der steigenden Prävalenz neurodegenerativer Erkrankungen rücken Lebensstilfaktoren immer mehr in den Fokus. Epidemiologische Studien deuten darauf hin, dass Pestizide das Risiko für α-Synukleinopathien wie PD und möglicherweise auch DLB erhöhen könnten. Die Pestizid-assoziierte PD ist in Deutschland bereits als Berufskrankheit anerkannt. DLB-spezifische Kohortenstudien in Bezug auf eine Pestizidexposition bestehen noch nicht, allerdings konnte ein Zusammenhang zwischen Pestizidexposition und einem erhöhten Risiko für eine RBD gezeigt werden 19 , welche als spezifischstes Prodrom für eine spätere α-Synukleinopathie (DLB, PD und Multisystematrophie [MSA]) gilt. Auch war in einer prospektiven Studie eine mediterrane Ernährungsweise mit einem um rund 10% verringerten kombinierten Risiko für PD und DLB assoziiert 20 , während ein hoher Konsum von ultrahoch verarbeiteten Lebensmitteln in einer Meta-Analyse zu einen Risikoanstieg von 44% für Demenzen aller Ätiologien führte 21 . Wiederholte leichte Kopftraumata, z. B. im Rahmen von Kontaktsportarten, sind ebenfalls mit einer erhöhten Prävalenz der PD und der DLB assoziiert 22 .

### Krankheitsverlauf

Studien zum natürlichen Verlauf sind essenziell, um die DLB umfassend zu charakterisieren und somit u. a. die Grundlage für zukünftige Therapiestudien zu legen. Es konnte gezeigt werden, dass der Krankheitsprozess der DLB häufig viele Jahre vor dem Auftreten klassischer Symptome beginnt. Mittels FDG-PET konnten metabolische Veränderungen bereits im prodromalen Stadium identifiziert werden, welche eng mit der klinischen Verschlechterung korrelieren 23 . Die Ausprägung motorischer Symptome, gemessen am MDS-UPDRS-III, war in einer aktuellen Studie bei DLB zwar insgesamt vergleichbar mit PD/PDD, jedoch unterschied sich das motorische Profil: DLB-Patienten zeigten weniger Tremor, stärkeren Rigor und Bradykinese sowie häufiger Gang- und Standunsicherheiten. Nicht-motorische Symptome, wie autonome Dysfunktionen, neuropsychiatrische Symptome und Schlafstörungen, scheinen bei der DLB deutlich häufiger aufzutreten als bei PD oder PDD. Retrospektive Langzeitdaten deuten darauf hin, dass AChE-I im Gegensatz zu Memantine den kognitiven Abbau verlangsamen sowie die Mortalität senken könnten 24 . Frauen erhalten im Vergleich zu Männern häufiger zunächst eine Fehldiagnose, was sowohl an der höheren Rate an AD-Kopathologie als auch an der weniger typischen Symptomkonstellation liegen könnte. Während Männer bereits früh 2–3 Kernsymptome, wie eine RBD, kognitive Fluktuationen oder Parkinson-Symptome, entwickeln, treten diese bei Frauen im Durchschnitt später auf 25 . Männer mit DLB weisen zudem eine früher einsetzende Hirnatrophie auf, was mit dem früheren Auftreten kognitiver Defizite und visueller Halluzinationen korreliert. Frauen erreichen im Verlauf jedoch mit ca. 75 Jahren eine ähnliche Ausprägung kognitiver Einbußen wie Männer 26 .

### Outcome-Parameter

Für den Erfolg klinischer Studien bei DLB ist die Auswahl geeigneter Outcome-Parameter entscheidend. Bisher werden überwiegend Testverfahren eingesetzt, die ursprünglich für andere Erkrankungen entwickelt wurden, sodass eine Validierung für DLB bzw. die Entwicklung DLB-spezifischer Skalen dringend erforderlich ist. Insgesamt sind die Outcome-Parameter bei klinischen Studien für DLB wenig standardisiert, was die Vergleichbarkeit erschwert. In einem mehrstufigen Verfahren unter Einbeziehung von Betroffenen, Angehörigen und medizinischen Experten wurden kürzlich acht Symptomkomplexe/Domänen definiert, die künftig als Mindeststandard für die Erfassung von Symptomen in Studien dienen sollen 27 . Mittels Sensor-basierter Wearables könnten Schwankungen motorischer Symptome im Tagesverlauf kontinuierlich erfasst werden 28 . Zur Erhöhung der Objektivität und Verringerung der Interrater-Variabilität bestehen wissenschaftliche Bestrebungen, etablierte Scores wie den UPDRS digitalisiert zu erfassen. Dies könnte zudem die benötigte Fallzahl für klinische Studien reduzieren.

### Biomarker

Molekulare Biomarker werden künftig über die AD hinaus eine unverzichtbare Rolle für den frühen Nachweis neurodegenerativer Pathologien, die Beurteilung des Krankheitsverlaufs und die Erprobung potenziell verlaufsmodifizierender Therapien spielen. In Bezug auf die Synukleinopathien stellt der α-Syn-SAA einen großen Fortschritt dar, mit dem sich der biopathologische Prozess der Prion-artigen α-Syn-Aggregation *in vitro* nachweisen lässt. Hierbei wird biologisches Material, in der Regel Liquor, zu einer Reaktionsmischung gegeben, deren Kernkomponente ungefaltetes α-Syn als Substrat für mögliche Aggregationsreaktionen ist. Befindet sich fehlgefaltetes α-Syn im Liquor, bilden sich Aggregate, welche mittels eines fluoreszierenden Markers gemessen werden. Die Ergebnisse folgen einem binären System, entweder liegt eine α-Synukleinopathie vom Lewy-Typ vor oder nicht. Versuche aus der Fluoreszenzkinetik des SAA (z. B. Latenzzeit, Zeit bis Maximum, Fläche unter der Kurve) Rückschlüsse auf Krankheitslast und Schweregrad zu ziehen blieben bislang ohne verlässliche Reproduzierbarkeit. Ein neuer Ansatz ist die Bestimmung des *Lewy-Fold-Pathology* -Wertes: Hierbei wird die Probe schrittweise mit gesundem Liquor verdünnt und ihre Aggregationsfähigkeit getestet. Dies erlaubt eine indirekte Abschätzung der α-Syn-Aggregat-Last 29 . Während α-Syn-SAAs bei PD-Patienten eine Sensitivität zwischen 75 und 100% zur Unterscheidung von gesunden Kontrollen erreichen 29 , liegt sie bei der DLB bei nur etwa 72–75% 30 31 .

Auch nuklearmedizinische Verfahren gewinnen zunehmend an Bedeutung 32 33 . Während die Amyloid- und Tau-PET-Bildgebung bei der AD bereits etabliert ist, steht derzeit noch kein PET-Tracer zur Verfügung, der eine Lewy-Typ α-Synukleinopathie zuverlässig nachweisen kann. Die Entwicklung eines Tracers gestaltet sich technisch unter anderem deshalb schwierig, weil α-Syn-Aggregate bei DLB und PD in deutlich geringerem Ausmaß vorliegen als z. B. Aβ und Tau bei der AD. Bereits eine leichte off-Target Bindung, z. B. an Myelin oder an andere aggregierte Proteine im Rahmen einer Kopathologie, kann das Ergebnis wesentlich beeinträchtigen, weshalb die Tracer ausgesprochen selektiv sein und über eine hohe Bindungsaffinität verfügen müssen.

### Diagnosekriterien

Um eine möglichst frühzeitige Diagnose zu ermöglichen, wurden zu wissenschaftlichen Zwecken die Diagnosekriterien einer prodromalen DLB vorgeschlagen 34 . Diese unterscheiden sich von den DLB-Kriterien dadurch, dass die Diagnose bereits bei einer leichten kognitiven Störung vergeben werden darf. Gerade im Kontext von Therapiestudien sind jedoch einheitliche, biologisch definierte Diagnosekriterien essenziell. Während bei AD die A/T/N-basierte biologische Definition 14 bereits weitgehend klinisch etabliert ist und eine Voraussetzung für Studieneinschlüsse darstellt, liegen für α-Synukleinopathien bislang nur rein wissenschaftlich konzipierte Vorschläge vor: Die SynNeurGe (Synuklein/Neurodegneration/Genetik)-Kriterien 35 und die *Neuronal α-Synuclein Disease biological definition and Integrated Staging System* (NDS-ISS)-Kriterien 36 . Beide Klassifikationssysteme basieren auf dem Nachweis pathologischer α‑Syn-Aggregate, dem Nachweis einer typischen Neurodegeneration (z. B. DaT-Scan, MIBG-SPECT etc.) und dem Vorhandensein einer vollständig penetranten oder stark prädisponierenden genetischen Variante (bei der PD deutlich häufiger als bei der DLB). Obwohl für beide Systeme bisher keine longitudinalen Daten zur Validierung vorliegen, und sie aktuell noch nicht in der Praxis zur Patienten-Stratifizierungen angewendet werden, stellen sie einen wichtigen ersten Schritt hin zu einer biologischen Krankheitsdefinition *in vivo* dar. In Bezug auf die DLB ist zu erwähnen, dass beide Klassifikationssysteme primär auf die PD ausgerichtet sind und die für die DLB hoch relevanten Kopathologien kaum berücksichtigen 31 .

### Therapiestudien

Obwohl DLB-spezifische Therapien dringend benötigt werden, konzentriert sich die Mehrheit der Therapiestudien für Synukleinopathien auf die PD oder MSA. Dennoch gibt es einige wenige Studien, die sowohl symptomatische Behandlungen als auch krankheitsmodifizierende Therapien für die DLB untersuchen.

Im Bereich der symptomatischen Therapien befindet sich aktuell der 5-HT₂A-Antagonist Remlifanserin zur Behandlung der DLB-assoziierten Psychose in einer multizentrischen, Placebo-kontrollierten Phase-2-Studie (NCT07029581). Das Vorgängerpräparat Pimavanserin ist in den USA für PDD-Psychose zugelassen und wird dort auch bei DLB angewandt. Bezüglich motorischer Symptome konnte der allosterische Dopamin-D1-Agonist Mevidalen (LY3154207) in einer Phase-II-Studie die motorischen Scores bei der DLB signifikant verbessern, jedoch ohne die primären kognitiven Endpunkte zu erreichen 37 . Weiterhin wird in einer monozentrischen Phase-IIa-Studie die Sicherheit und Wirkung des Natrium-Kanal-Inhibitors AR1005 zur Behandlung von kognitiven und psychischen Symptomen, Fluktuationen und motorischen Symptomen bei der DLB untersucht (NCT06537076).

Auch werden erste krankheitsmodifizierende Therapieansätze bei Lewy-Typ α-Synukleinopathien in klinischen Studien erprobt. Das *small molecule* Neflamapimod, ein oraler p38α-MAP-Kinase-Inhibitor, welcher durch Hemmung neuroinflammatorischer Signalwege potenziell neuroprotektiv wirkt, führte in einer explorativen Phase‑IIa-Studie bei der DLB zu signifikant besseren kognitiven und motorischen Outcomes gegenüber Placebo 38 . Die Effekte waren am deutlichsten bei Patienten ohne AD-Kopathologie. Basierend darauf wurde das Phase‑IIb-Studienprotokoll RewinD-LB (NCT05869669) angepasst und Patienten mit nachgewiesener AD-Kopathologie ausgeschlossen 38 . Endgültige Ergebnisse der RewinD-LB-Studie stehen noch aus. Ein weiterer potenzieller Therapieansatz umfasst die Reduktion von α-Syn. In einer Phase-II-Studie bei der DLB wurde der Tyrosin-Kinase-Inhibitor Bosutinib gut toleriert und führte im Liquor sowohl zu einer Reduktion der α-Syn-Konzentration als auch der Dopamin-Abbau-Produkte 39 . Eine größere Wirksamkeitsstudie steht jedoch noch aus. Die laufende Phase-IIa-Studie ANeED (NCT04588285) prüft die Wirkung des Chaperons Ambroxol auf die Kognition, das Funktionsniveau und neuropsychiatrische Symptome bei der DLB. Ambroxol ist ein zugelassenes Mukolytikum, welches *in vitro* die GBA-Aktivität erhöhen konnte und somit den α-Syn-Abbau steigerte 40 . Weitere, in klinischer Erprobung befindliche, Mechanismen mit Potential zur Verlaufsmodifikation von Synukleinopathien und damit auch von der DLB sind z. B. α-Syn-gerichtete Vakzinierungsstrategien oder *small molecules* als Hemmer pathologischer Aggregation 41 42 . Diese könnten in der Zukunft auch bei der DLB erprobt werden.

## Fazit für die Praxis

An eine DLB sollte insbesondere bei Patienten mit einem demenziellem Syndrom mit exekutiven und visuokonstruktiven Einbußen in Kombination mit oftmals subtilen Parkinson-Symptomen gedacht werden. Ebenfalls typisch für die DLB sind Fluktuationen der Wachheit und Kognition, ausgeschmückte visuelle Halluzinationen und eine RBD. Die Diagnosestellung erfolgt anhand der McKeith-Kriterien 34 , welche klinische Kriterien und Biomarker, wie z. B. den DaT-Scan, kombinieren. Die Therapie erfolgt rein symptomatisch, z. B. mit AChE-I zur Verbesserung der Kognition oder Levodopa bei motorischen Symptomen, und sollte individuell nach Symptomlast und Verträglichkeit angepasst werden. Von „klassischen“ Antipsychotika wie Haloperidol ist aufgrund des Risikos einer lebensbedrohlichen akinetischen Krise auch bei ausgeprägten visuellen Halluzinationen unbedingt abzusehen. Eine mögliche und wirksame Alternative stellt Clozapin dar.

## Notes

Abkürzungen
**ARTAG** *ageing-related tau astrogliopathy*
**AChE-I** Acetylcholinesterase-Inhibitoren
**mTL** medialer Temporallappen
**SNRI** Selektiver Serotonin/Noradrenalin-Wiederaufnahmehemmer
**SSRI** Selektiver Serotonin-Wiederaufnahmehemmer
**DLB** Demenz mit Lewy-Körperchen
**PDD** Parkinson-Demenz
**RBD** REM-Schlaf-Verhaltensstörung
**PD** Parkinson-Krankheit
**α-Syn** alpha-Synuklein
**LB** Lewy-Körperchen
**AD** Alzheimer-Krankheit

**TDP-43** *Transactive response DNA-binding protein 43*
**FTLD** frontotemporale Lobärdegeneration
**APOE ε4** Apolipoprotein E ε4
**GBA** Glukozerebrosidase
**SN** Substantia nigra
**PSG** Polysomnographie
**DaT-Scan** Dopamintransporter-Szintigraphie
**MIBG-SPECT** 123Iod-MIBG Myokard-Szintigraphie

**α-Syn-SAA** *alpha-Synuklein Seed Amplification Assay*
**Aβ** beta-Amyloid
**MSA** Multisystematrophie
